# “The Boundless Realm Where All Form Lies”. Representing Imagination at the Crossway Between Literary and Neurocognitive Studies

**DOI:** 10.3389/fnint.2020.618605

**Published:** 2021-01-29

**Authors:** Renata Gambino, Grazia Pulvirenti

**Affiliations:** ^1^Department of Human Studies, University of Catania, Catania, Italy; ^2^NewHums – Interdipartimental Center for Neurocognitive and Human Studies, Catania, Italy

**Keywords:** imagination, representation, embodied simulation, German eighteenth century culture, literature analysis, aestheticse

## Abstract

According to ancient texts on poetics, the concept of representation is deeply bound to that of “mimesis;” this last was intended in two main ways: as “imitation” and as “world construction.” In Aristotle's *Poetics*, mimesis is theorized as the main form of “world simulation,” giving rise to the complex universe of fiction. The concept of simulation plays a pivotal role in the neurocognitive theories on the embodied mind: within this frame, embodied simulation is intended as a functional prelinguistic activation of the human sensorimotor mechanism. This happens not only with regard to intercorporeality and intersubjectivity in the real world but also in relation to the process of imagination giving rise to literary imagery and to the reader's reception of the fictional world, since human beings share a common sensorimotor apparatus. Imagination is a central concept in the recent neurocognitive studies since it plays a core role in human life and in artistic production and reception. Imagination has been considered as a complex emergent cognitive faculty deeply intertwined with perception, memory, and consciousness, shaping human life and transforming the limited horizon of our perceptual affective understanding, being, and acting. Although there is an immense bulk of literature on this topic, imagination is still an elusive concept: its definition and understanding change according to different heuristic frames—mainly the philosophical, aesthetic, poetic, and cognitive ones—giving rise to debates about its modalities and effects, particularly in relation to the construction of aesthetic and symbolic constraints. In this paper, we claim that scientific research may take advantage from the literary representation of the imaginative faculties, which occurs in specific tests characterized by dynamic images and motion. In such meta-representation of the imagination, we witness the phenomenological emergence of endogenous dynamic processes involving a cluster of cognitive faculties, activated by triggering the reader's embodied simulation. One of the main German poets, Johann Wolfgang von Goethe, in the second part of his masterwork *Faust II*, intuitively represents the very process of the imagination and its responding to embodied simulation with regard both to the author's creative act and to its reception by the reader. At the crossway between literary and neurocognitive, this study aims to highlight the advantage offered to future transdisciplinary inquiries by the literary representation showing features and dynamics of the still mysterious phenomenon of the imagination.

## Introduction

And as imagination bodies forth

The forms of things unknown, the poet's pen

Turns them to shapes and gives to airy nothing

A local habitation and a name.

Shakespeare, *A midsummer Night's dream* (act V, scene 1, 1844–1846)

According to ancient texts on poetics, the concept of representation is deeply bound to that of “mimesis.” This last was intended in two main ways: as “imitation” and as “world construction.” In Aristotle's *Poetics*, mimesis is theorized as the main form of “simulation” in the sense of “world construction,” giving rise to the complex universe of fiction. The concept of simulation has been developed in the groundbreaking researches by Maturana and Varela (1985, 1987) and in recent neurocognitive studies, specifically in the 4E Cognition avenue, i.e., within the theories regarding cognition as embodied, embedded, enacted, and extended (Newen et al., 2018). In this heuristic frame, a core process is that played by the activation of the “embodied simulation,” which is to be intended as a functional prelinguistic activation of the human sensorimotor system (Gallese, 2019). This mechanism is active in our life in the real world, giving rise to all phenomena bound to intercorporeality and intersubjectivity (empathy, sympathy, compassion, social cognition, etc.) (see Gallese, 2005, 2007, 2011, 2016; Freedberg and Gallese, 2007; Gallese et al., 2009). According to recent studies, the same system is reused in all imaginative processes particularly in those presiding over the creation and reception of artworks, theatrical performances, and literary texts (see Cook, 2010; Gosetti-Ferencei, 2018). Although it is one of the most investigated processes of the human mind, imagination has been addressed in a breadth of different perspectives, leading to a manifold of interpretations as well as evaluations. Many hypothesis and theories highlight different features and behaviors of the imagination (for review, see Brann, 1991; Kearney, 1998; Stevenson, 2003); some of them regard it as regulating the relation among percepts and mental representations in the construction of the “human imagination spectrum” (McGinn, 2004) as well as the “multidimensional continuum view” (Thomas, 2014), determining the image of the world surrounding each perceiving subject.

At the crossway among presentation, representation, and mental imagery, the imagination plays a significant role not only in “representing” to the “mind's eye” the given but also what does not exist in reality and is evoked out of the sphere of pure potentialities of the “invisible” (Franzini, 2001). This creative aspect has been the object of sophisticated literary representations of the very process of the imagination, modeling the visible and the invisible. In fact, the literature may be considered as a sort of symbolic stage, where we may discover the most refined inferential and representational mechanisms, which preside over the creation of a counterfactual world of inexhaustible images (Gambino and Pulvirenti, 2019a,b). More specifically, we will show our hypothesis on the dynamics of literary texts representing imagination at work, by analyzing some passages from Johann Wolfgang von Goethe's masterwork *Faust II—*the controversial scene *Gloomy Gallery* and *The Hall of the Knights, Dimly Lit* (act I, scenes V, VII)—representing imagination along its ontogenetic and phylogenetic development. The metaphorical quality of these scenes anticipates some aspects of the imaginative processes partially highlighted by the recent neurocognitive sciences, evidencing its being an endogenous, dynamic, emergent process involving a cluster of cognitive faculties activated in order to construct meaning through the creation of aesthetic forms. Goethe intuitively represents the very process of the imagination and its responding to embodied simulation with regard both to the author's creative act and to its reception by the reader.

This study will refer to a transdisciplinary methodological frame at the epistemic convergence of literary and neuroscientific approaches, bridging results from neurocognitive theories on embodied simulation, as well as from cognitive literary criticism and neurohermeneutics (Gambino and Pulvirenti, 2018, 2019a,b). Our final goal is to highlight how language may mediate the representation of the imagination activating embodied simulation processes leading to the creation of counterfactual worlds and their reception.

## Methodological Premise

In the prosecution of Maturana's and Varela's research (1985, 1987) and within the recent heuristic context of the “4E Cognition” (Newen et al., 2018), the nature of the mind is considered as embodied, embedded, enactive, and extended, with regard to the coupling of brain, body, and environment. Therefore, the role of action has become a core issue within the studies on perception, intentionality, empathy, social cognition, and culture production. Within the “virtual space” of fiction, the representation of literary imagery mirrors the complex dynamics that the author carries out in the elaboration of his/her own experience of the world, creating a complex “device” (the literary text), in which the mental faculties and functions that characterize the human as a sentient, conscious, and knowing being, become manifest as on a stage, mirroring themselves within an implicit mind's dialogue among the author, the text, and the reader. The specific images arising from each unique literary representation trigger the reader to imagine, emotionally feel, and cognitively get meanings out of the own imaginative elaboration of the literary images “embedded” in the formal features of the literary text—such as language, style, and rhetoric figures. The pivot of this process is the imagination, which presides over the creation of counterfactual worlds, on their turn triggering the readers' imagination to recreate an inexhaustible world of mental images resonating the textual ones (Gallese, 2018b).

These issues show multiple implications not only in the research fields of cognition, evolution, social behaviors, subjectivity, and empathy but also in the study of the imagination (see Gosetti-Ferencei, 2018). Among the manifold of the modes of the imagination, we will here consider it as an embodied emerging process significant to any human experience reflecting on and transforming the world, either in thought, and/or in cultural products, such as fine arts, literature, performance, and multimedia products. Imagination turns out to be intertwined within the embodied life “through the examples of explicitly embodied imagining in performance art, dance, and the making of film, as well as the evocations of embodiment through painting, literature, and social responsiveness” (Gosetti-Ferencei, 2018, p. 23–24). Its literary representation becomes a paradigmatic exposure of the phenomenology of the imagination as one of the most complex and surprising cognitive embodied achievements of the human mind.

### Embodied Simulation

The intertwining among cognitive processes and physiological activations, summarized in the concept of the embodied mind, was the core issue of Humberto Maturana's and Francisco Varela's revolutionary studies *Autopoiesis* (1985), and *The Tree of Knowledge* (1987), investigating the embodied mechanisms of creation, self-regulation, and cognition of living forms from a biological perspective (see also Rudrauf et al., 2003). In the autopoietic organization, being, operating, and knowing coincide in a network of continuous dynamic interactions of the molecular components of each cell unit. The cellular metabolism produces such components that integrate the network of transformations from which they were produced and further through metabolic dynamics forming themselves as distinct elements, all within the surrounding environment (Maturana and Varela, 1985, p. 62). Multicellular living beings are characterized by the somatosensory and motor components and the mechanisms of their dynamic relationship in the nervous system, whose architecture is universal but whose complexity and amplitude changes in the diverse living beings. According to Varela and Thompson, such active intertwining is inscribed in the concept of “double embodiment” of the mind in the body and of the latter within the environment:

By using the term *embodied* we mean to highlight two points: first that cognition depends upon the kinds of experience that come from having a body with various sensorimotor capacities, and second, that these individual sensorimotor capacities are themselves embedded in a more encompassing biological, psychological and cultural context (Varela et al., 1991, p. 172–173).

The complex phenomenon of cognition derives from the interaction among an individual brain–body system and that of others: “To make sense of cognition we need to study the brain, the body, their relationship with the world and with the brain-body systems of others” (Gallese, 2018a, p. 31). Cognition is considered as a form of embodied experience of the human being in the world. This means that knowledge is no longer understood as a result of formal operations of abstract symbols but as enaction (Varela et al., 1991; see also Noë, 2004 for the link between perception in an enactivist view), i.e., as action generated from within the body in the process of its interaction with the world, culminating in the creation of a complex system of actions and meaning construction. Perception, experience, and cognition arise from the relationships that are created among the embodied mind and the environment in which the body is located and within which it interacts. Therefore, a basic phenomenon, such as that of perception, does not occur passively, but it is the result of an action changing on the basis of the changes that the nervous system undergoes in the experience of the world: the same sensorimotor embodied circuitries change during the implementation of activities (Thompson, 2001, 2007; Gangopadhya et al., 2010; Noë, 2010).

According to Vittorio Gallese (2000, 2003, 2005, 2009, 2011), human beings share a common sensorimotor apparatus, that is, at the basis of both the phenomenon of intercorporeality in the real world and of embodied simulation presiding also over the creation of art and its reception, like in the case of the embodied reading act. In the first case, it contributes to the phenomenon of intersubjectivity, that is, the social capacity to understand actions, motor intentions, sensations, and emotions of others; in the second case, it is active during the interaction of the reader with the verbal text in its various components: the images, the linguistic, and rhetorical texture of a text, actions, events, and characters of the fictional world.

Starting from the experience of the body in relation to the environment, the human brain maps reality, according to the bodily ability to act and to move in space. This creates a sort of neural dictionary of actions that structures and articulates our relation with the world in which we are immersed. That is to say: we read the world according to our physiological potentialities and abilities to interact with it, constructing hierarchical representations of actions and motor goals, thanks to our shared motor system, which is organized according to goal directed motor acts (see Gallese, 2000, 2009, 2014, 2016, 2018a; Gallese et al., 2009; Gallese and Cuccio, 2015).

Part of the phenomenon described as motor cognition is based on motor simulation, whereby cognitive abilities, such as the identification of motor purposes in the behavior of others, as well as the anticipation of actions, are possible because of the functional architecture of the motor system, which is organized in terms of motor actions with specific purposes. In the words of David Freedberg and Vittorio Gallese:

Our capacity to pre-rationally make sense of the actions, emotions and sensations of others depends on embodied simulation, a functional mechanism through which the actions, emotions or sensations we see activate our own internal representation of the body states that are associated with these social stimuli, as if we were engaged in a similar action or experiencing a similar emotion or sensation. Activation of the same brain region during first- and third-person experience of actions, emotions, and sensations suggests that, as well as explicit cognitive evaluation or social stimuli, there is probably a phylogenetically older mechanism that enables direct experiential understanding of objects and the inner world of others (Freedberg and Gallese, 2007, p. 198).

Gallese claims that we elaborate a representational content of what we see and interact with on the basis of our ability to bodily simulate the purposes of actions and of the objects' affordances. In fact, embodied simulation relies on the reuse of the sensorimotor system that, during the evolution, has been decoupled from motor purposes in the real world and reconnected with other cortical areas recalling its activation:

Individuals reuse their own mental states or processes in functionally attributing them to others (Gallese, 2009, 2011; Gallese and Sinigaglia, 2011). The extent and reliability of such reuse and functional attribution depend on the simulator's repertoire and its being shared with the target's repertoire. Brain and cognitive resources typically used for one purpose are reused for another purpose. For example, witnessing someone else expressing a given emotion (e.g., disgust, pain) or undergoing a given sensation (e.g., touch) recruits some of the visceromotor (e.g., anterior insula) and sensorimotor (e.g., second somatosensory area, SII; ventral premotor cortex) brain areas activated when one experiences the same emotion (Gallese, 2011, p. 197).

### Embodied Simulation, Imitation, and Literary Language

The concept of the embodied mind was complemented by that of extended mind to indicate that mental processes do not reside only in brain activity but are extended to artifacts and social configurations that involve the reality of the mindbrain modifying its skills and knowledge. In this perspective, also language is to be regarded as a result of an interaction among organisms, while the vast field of cultural phenomena is considered as product of a transgenerational stability of behavioral configurations acquired ontogenetically in the dynamics of communication within a specific social and cultural environment (Lakoff and Johnson, 1980, 1999; Maturana and Varela, 1985, p. 170).

Starting from these premises, the “simulative theory” of language, already hypothesized by Varela, attests that the linguistic activity is to be attributed to the same brain areas used for movements, since simulative mechanisms are anchored in our corporeity, in a “model of bodily representation” (Cuccio et al., 2013, p. 90). By listening to utterances indicating actions, a brain process of simulation of that action is carried out: this means that language implies the activation of the motor system, for which Gallese concludes that through reading or listening to a sentence describing an action, the motor representation of the same action is activated. Such a motor activation can take place also with regard to abstract language or to the figurative use of language, as it happens in the case of metaphors: the activation of the simulation process in the linguistic comprehension suggests that the symbolic dimension and the corporeal dimension cohabit in the linguistic praxis.

Embodied simulation has also been addressed to with regard to the gestural origin of language (see Castelli et al., 2006), asserting a continuity between prelinguistic and linguistic expressions, i.e., between actions, gestures, and words depending on the motor cortex and involving part of the neurons responsible for hand and mouth control. Broca's area presides over the acquisition of language (see: Arbib, 2006, p. 19), which mainly involves the same brain areas where mirror neural systems seem to be more widespread and in interaction with the motor areas. This suggests that executing an action, observing it, listening to it, or reading the linguistic description of such action induces a motor simulation that activates some of the same regions of the cortical motor system. In fact, there is a close anatomical and functional relationship between action and semantics, that is, a sensorimotor integration between the action of the subject in relation to an object and its meaning. In fact, some areas of the brain (including the frontal, parietal, and temporal ones) produce something like a *copy* of the motor patterns in order to perform actions with respect to things in the world and in relation to the coding of meaning in reality (Gallese et al., 1996). According to the concept of embodied simulation, as Vittorio Gallese and Valentina Cuccio pointed out, the common denominator given by the physical body and its characteristics allows to infer emotions and moods also through reading:

Compelling evidence shows that humans, when processing language, activate the motor system both at the phono-articulatory and at the semantic level. When listening to spoken words or looking at someone speaking to us, our motor system simulates the phono-articulatory gestures employed to produce those very same words. Furthermore, processing action-related linguistic expressions activates regions of the motor system congruent in somatotopic fashion with the processed semantic content. Reading or listening to a sentence describing a hand action activates the motor representation of the same action (Gallese and Cuccio, 2015, p. 11).

This perspective changes radically our way of regarding literary representations and imagery, since it sheds new light on the pivotal role played by the sensorimotor engagement of our body in the aesthetic experience and in the linguistic production and reception:

The activation of motor representations in the brain of the reader or listener has been demonstrated at the phono-articulatory level, as well as during the processing of action-related linguistic expressions (words and sentences) and of morpho-syntactical aspects of language. This evidence, although widely discussed, points to a causal role of embodied simulation in language processing and understanding (Gallese, 2011, p. 198).

The “simulation theory” of language claims that linguistic activity is to be attributed to the activation of cerebral areas used for perception and movements. The simulative mechanisms are bodily anchored in a model of bodily representation. Simulation happens, thanks to a mimetic stance, which makes use of the neural correlation of sensory, perceptual, and motor functions, involving not only the observation of facial and bodily emotional expressions but also the Broca's area, which is the motor area for speech and language, as Iacoboni pointed out:

[…] Broca's area is the motor area for speech, and learning by imitation plays a crucial role in language acquisition. […] Language perception should be based on a direct matching between linguistic material and the motor actions responsible for their production. Broca's area is the most likely place where this matching mechanism might occur (Iacoboni et al., 1999, p. 2527).

This implies that seeing someone perform an action or listening or reading the linguistic description of an action can induce a motor simulation that activates some of the same areas of the cortical motor system, including those with mirror properties (Iacoboni et al., 1999). The sensory and motor activations are fundamental not only to linguistic comprehension but also to the linguistic elaboration of our complex bodily experience, both in terms of subjective experience and conceptualization. The link between the sensorimotor system and abstract thought would be provided, according to some cognitive linguists (Lakoff and Johnson, 1980; 1999) by image schema, which would be a hinge between the embodied world and the conceptual world:

What Johnson and Lakoff have called *image schema* are precisely these patterns of sensorimotor experience that are permanently recurring, through which we interact with the environment we understand and within which we act to realize our intentions. Numerous studies conducted in different fields, from experimental psychology to linguistics to developmental psychology, confirm the existence of these image patterns. According to our hypothesis, these image patterns are embodied at the neural level as activation patterns in the space of our topological neural map. Image patterns are therefore part of our non-representational connection with our world, in the same way that barnacles and squirrel monkeys have image patterns that define the different types of their sensorimotor experience. The structure of *image schema* is the basis of our ability to understand all aspects of our perception and motor activities (Johnson and Rohrer, 2007, p. 28).

Language's production and understanding rely on the activation of our sensorimotor system and evolve through functional conceptualizations, establishing interactions with the world (*image schema*). This process allows the human being to produce a simulation of his/her interaction with the environment at the level of the imagination. The progressive development of this capacity of abstraction, which is inherent to language, on the one hand, presides over the creative imagination of a counterfactual world activating simulation processes. These, on the other hand, solicit in the reader *mental imagery*, i.e., a vivid experience simulated by the reader's mind in the absence of corresponding physical stimuli (see: Kuzmičová, 2013, 2014), similarly to those experiments eliciting sensations and “natural” percepts via the brain electrical stimulation, producing unnatural or natural perceptions or movements, even without stimulation of peripheral sensory receptors (Armenta Salas, 2018). In neurological terms, the very act of reading is related to a production of mental images through the activation of the extrastriate visual cortex (Zull, 2002, p. 169), whose area V5/MT is sensitive to processing visual motion.

All in all, embodied simulation may be considered as a mechanism both presiding over and giving access to the fictional world of literature: the same mechanism of simulation that allows an author to elaborate bodily experiences and emotions in images mediated by stylistic and rhetorical figures, characters, and motifs allows the reader to access the fictional world, relating it to the own experiential horizon. In this sense, Gallese, together with Hanna Wojciehowski, consider embodied simulation as “the outcome of a basic functional mechanism instantiated by our brain–body system, enabling a more direct and less cognitively-mediated access to the world of others and to […] fictional narratives (Wojciehowski and Gallese, 2011, p. 7). Gallese underlines the similarities between the aesthetic experience and real life, both relying on the activation of embodied simulation, triggering an effective emotional response that appeals to the reader in a more or less strong way, depending on the typologies of specific characteristics of narration, images, and stylistic and rhetorical figures:

Hence, embodied simulation theory can be used to both account for how we perceive the world and how we imagine it and build a world of fiction and experience it. My hypothesis is that the world of cultural artifacts is “felt” not too differently from how we feel the more prosaic world we encounter in daily life. We feel for and empathize with fictional images and characters in ways that are similar to how we feel for our real social partners, although with qualifying differences (Gallese, 2018a, p. 117).

Further studies have investigated narratives and narration in relation to embodied theories, underlying the role of action in the imaginative production of narration (Armstrong, 2017; Cave, 2017) (GP).

### Imagination

For the core role played in the human life, imagination is a central topic in the latest neurocognitive research. Many scholars, like Mark Turner, have stressed the epistemic need of cognitively investigating the processes of imagination not only in order to improve our understanding this great mystery of the human mind but also to explain some other faculties of the human brain and mind system relying on imagination (Turner, 2000). All in all, imagination is intended in the immense of its activities as a complex emergent cognitive faculty deeply intertwined with perception, memory, and consciousness, shaping the human life and transforming the limited horizon of our perceptual affective understanding, being, and acting.

Although there is an immense bulk of literature on this topic, imagination is an elusive concept more frequently referred to than explained, even by philosophers—from Aristotle to Hume, from Kant to Husserl. Its definition and understanding change according to different heuristic frames—mainly the philosophical, aesthetic, poetic, and cognitive ones. Different discourses on imagination have given rise to still unsolved questions about its modalities and effects, particularly in relation to the construction of aesthetic and symbolic constraints: they derive from our imaginative abilities to regard and manipulate things and experiences of the real world through the wide spectrum of their unexpressed potentialities, even transcending the given ones. The imaginative manipulation of reality involves the embodied activation of our sensorimotor system, which is reused when the mindbrain processes counterfactual images, elaborates them through language and different media, or when it is receptive to mental imagery (Kuzmičová, 2013, 2014) triggered by linguistic and visual artworks.

During the “iconic turn” and even earlier in political studies during the 1970's, imagination was depreciated as Huppauf and Wulf pointed out:

In the aftermath of the radical sixties, skepticism toward the social and political power of the imagination became popular. A political interpretation of the imagination created great expectations but they, it turned out, were mistaken. […] The imagination has had an uneven and controversial history in the modern period. For most of the time it was considered secondary and ancillary and sometimes even a dangerous human faculty. […] It was placed among the weaknesses of the human nature and, at the same time, seen as an origin of the fear of losing control over reality. It seemed irreconcilable with the ideal of self-determination through the production of knowledge in scientific disciplines (see Huppauf and Wulf, 2009, p. 1–2).

The pivotal volume edited by the two above-mentioned scholars had the advantage of “re-assessing the role of the imagination for theorizing the return of images in the historical moment of the pictorial turn.” (Huppauf and Wulf, 2009, p. 4). On the contrary, in the neurocognitive endeavors of the last century, in evolutionary anthropology, as well as in cognitive and developmental psychology, the imagination has never ceased to be investigated as a fundamental representational activity of our consciousness: it enables human beings to shape in a creative way their own experience, perception, and vision of the self and the world, as well as memories and emotions, manipulating and transforming them into counterfactual images, thanks to the ability of reusing the somatosensory and motor system (Gosetti-Ferencei, 2018; Gallese, 2019).

One of the main controversial issues in the debate about the imagination, which was raised in ancient Greek philosophy, and has been significant to the Western thought of the last century, regards its “presentative” quality, i.e., the capacity “to make present” in mental imagery or in cultural artifacts what is present to consciousness (Gosetti-Ferencei, 2018). Mental or artistic images differ deeply from the perceived ones, since the lived experience is itself a subjective generative transformation of what we sense and interact with. This issue requires a fundamental distinction among reproductive and productive imagination. This distinction was crucial in Kant's theories and in widespread discourse on imagination in the aesthetics of the 18th century. For Kant, imagination defines, on the one hand, the process compressing multitude and multiplicity into mental images and into meaningful symbols and, on the other hand, the faculty of expanding and transcending human experience over its cognitive and perceptive limits (Kant, 1781, § 10).

For Kant, the imagination is the cognitive ability of combining and mentally elaborating counterfactual images, according to associative, rational, or synthetic rules. Productive imagination [*Einbildungskraft*] binds different images [*Vorstellungen*] together, following the rules of reason, structuring them on the basis of pure concepts (categories). The synthesis produced by the imagination is therefore to be considered as a product of reason depending on perception, achieving a unitary representation of the world, bringing “the plurality of perceptions into an image,” and endowing it with meaning (Kant, 1781, A120, B179). In this perspective, the imagination intervenes as a principle of unification in any perceptual process and in any interpretative act, transforming our experience of the world inside and outside us in meaning constructions. Therefore, Kant considers the imagination as the faculty able to connect all scattered elements gathered by human perception into a cognitive, memorial, and emotional productive synthesis. Definitively, the imagination is regarded by Kant, to whom Goethe draws back, as a mental faculty that operates in two different modalities: as “reproductive” power of recreating in the mind perceptual experiences and objects and as a “productive” one, which evocates in the eye–mind visions of objects that are not present.

Also Goethe distinguishes between a “mimetic” (reproductive) and a non-mimetic “productive” form of imagination. This issue is well-explained in Goethe's letter to Karl Ludwig von Knebel (21. February 1821):

Observation needs imagination: imagination works at first *imitating*, just replying things; then it becomes *productive*, giving life to the comprehended, developing it, expanding it, transforming it. Further we think there is another form of imagination: the *enlightening* one, looking around on evidence, capturing similes in order to confirm the defined.

Great is the power of analogy, applied by the spirit to so many important elements, and powerful the way it works by putting together what belongs together or what seems similar.

This is the way we create *similes*. The closer to the object they are, the worthier and enlightening. The best of them are those that seem to cover it up completely and to be identical with it (Goethe, 1887-1919, 4.34, 131).

The “mimetic” imagination reproduces the external world of phenomena and objects in an analogous “imitating” way; the “productive” allows to implement and empower the perception of the real world applying to language the same laws ruling on nature (development, expansion, transformation). The first one relies on the principle of reproduction (*Nachbildung*) and the second on the principle of animation (*Belebung, Ursprung des Lebendigen*); both principles are derived from his morphological studies (Goethe, *Zur Morphologie*, WA, II, 6). Interestingly enough, Goethe puts out a third modality of imagination, considered as the most “revealing” one: the *enlightening* imagination (Goethe, 1887-1919, 4.34, 131). We use “enlightening” to translate the German term “umsichtig,” which refers to a sort of all-around gaze, the very gaze that precedes the moment of creative representation. This particular faculty of the imagination refers to the generative production of images relying on the biological principle of the living world: the human being senses everything scattered in parts but, through his/her imagination, has the intuition of the not manifest essence of the whole, which is not caught by human perception. Thanks to its creative power, the imagination “enlightens” the physical objects of the world by recovering their noumenic essence. Both phenomenal forms of nature as well as verbal images derive from the same infinite creative organic and transformative power inherent to nature (*Thatkraft*), which symbolic and metaphorical images are able to represent at conscious scale (Cohn, 1978).

Thanks to its creative power, the imagination is able to represent phenomenal events through words, since language participates in the organic complexity of the existence and shares the common morphological roots of the whole living system (Abel, 2011). In this perspective, the imagination is active giving form to perceptions, in order to submit experiences to logical procedures and to the laws of vital relations. In this sense, Goethe's “enlightening” imagination plays a significant role in joining the two attitudes of reproducing and producing images of the real world and counterfactual images in the arts and in literature (Goethe, 1887-1919, 32, 304).

### Representing Imagination

Interestingly enough, besides the intense scholarly debates in different heuristic discourses and epochs, the imagination has also been object of artistic and literary meta-representation: in such cases, it has not been discussed or reflected upon but directly phenomenologically represented as a productive force at work.

In this sense, we interpret Goethe's evocation of the Mother's Kingdom in the scene *Gloomy Gallery* in *Faust II*: here, Goethe goes beyond the limits of the human mind's sensory experience trying to represent the invisible, pre-noetic background of all possible forms, by imagining a dynamic, metamorphic, floating cloud full of all possible forms. Goethe tries here to overcome the cognitive limits of figuring the invisible, intended as a sort of precategorical dimension, a flurry horizon of pure, unexpressed potentiality—a realm of “possibility and latency” (Merleau-Ponty, 1967, p. 44): this realm includes all hidden elements, forcing vision to go beyond the threshold of visibility and matter. In this scene, Goethe represents the imagination as a complex dynamic emergent process, further “performing” its impact during the receptive act in the scene *The Hall of the Knights, Dimly Lit*.

The scene begins after a great allegorical parade at the Emperor's palace, when Faust is asked to bring on stage, in a sort of illusionary show, the prototype of beauty: “the ideal form of Man and Woman” (v. 6185). To carry out this request, Faust has to face a dangerous challenge. Mephistopheles warns him about the difficulties implied in getting the ideal form of beauty in front of the audience because this requires Faust to reach the pure indistinct potentiality of form itself, represented by a vortex, a turmoil of fluctuating images that perception is unable to identify and comprehend. This experience takes place in a mysterious and void landscape, the Kingdom of the Mothers, where no space nor time exist. This scene puts us in front of the fundamental question expressed by Merlau-Ponty and then reformulated by Thompson: “What is the mode or manner in which form appears to mind and what is the epistemological origin of this mode of giveness?” (Thompson, 2007, p. 81).

To imaginatively represent the “giveness” of the Mothers' Kingdom, Goethe tries to represent a “greater mystery” (6211), i.e., the imagination as a reign of pure potentiality and unexpressed form, characterized by the absence of all human cognitive categories, like space and time, limited extension, and sharpness:

Mephistopheles […]

Goddesses, enthroned on high, and solitary.

No space round them, not even time: only

To speak of them embarrasses me.

They are The Mothers! (6212–6215) […]

And if you'd swum through every ocean,

And seen the boundless space all round

You'd still have seen wave on wave in motion,

Though you might have been afraid to drown.

You'd have seen *something*. Seen, within

The green still seas, the leaping dolphin:

Seen clouds go by, Sun, Moon and star—

You'll see none in the endless void, afar,

Hear not a single footstep fall,

Find no firm place to rest at all (6239–6248).

In the following lines, Mephistopheles evokes the Mothers' realm as a great turmoil of clouds:

Into the boundless realm where all Form lies!

Delight in what's no longer on the list:

Where turmoil rolls along all cloudily:

Then, far from your body, swing the key! (6278–6281)

This “most alien sphere” (v. 6195) is just a vast and fluctuating cloud, full of contrasting and transforming images. This non-consistent reality, the “boundless realm of all forms” (6277), is represented by a metaphorical oxymoron tackling the two contrasting features of formal definition and boundless extension: “Into the boundless realm where all Form lies” (6278). The pre-noetic realm generating all forms, at the fringe of being and not being, is represented as a cloudy turmoil characterized by autonomous motion and directly put in relation to the same force governing and triggering human thought: “Formation, Transformation, / eternal minds eternal recreation” (6288). In order to access this most unimaginable reign, this vast and empty space, Faust has to find a glowing tripod, signaling his arrival in the “boundless realm of all forms” (6277). To guide Faust in his descent, Mephistopheles provides him with a “little key,” growing and glowing in his hand. Thanks to the key, Faust will be able to find the place where the tripod glows and to hook it, making the Mothers appear:

By its light [of the tripod] you'll see Mothers,

Some sit about, randomly, the others,

Stand and move. Formation, Transformation,

Eternal minds' eternal recreation.

Images of all creatures float, portrayed:

They'll not see you: they only see schemes (6285–6290).

In the very moment of their manifestation, Faust is able to perceive the pure potentiality of creation:

In your name, Mothers, you enthroned

In boundlessness, set eternally alone,

And yet together. All the Forms of Life

Float round your heads, active, not alive (6427–6434).

These mysterious goddesses are surrounded by a moving fluctuating essence without form but rich in pure imaginal metamorphic content. The floating image of clouds symbolizing the infinite transformation of nature recurs in Goethe's works generally as a metaphor of transformation (see for instance the poems *Ganymed* and *Howards Ehrengedächtnis*). Goethe's dynamic fluid meta-representation of the imagination through a process of transforming fuzzy images in turmoil, in which the imprecise perceptual issues are integrated and empowered by the quality of motion and instability, anticipates issues of the embodied conception of imagination (Gambino and Pulvirenti, 2013) as self-organized and emergent, implying the activation of multiple neural circuits, which are involved in the embodied simulation mechanism in real life. Representing imagination as a power of “formation” and “transformation” (6287), as linking different percepts and experiences through image schemas (the key and the tripod), giving form to noetic thought and meaning (“eternal minds eternal recreation,” 6288), Goethe represents the imaginative process as a force at work, an integrated and dynamic flow of sensorimotor, memorial, visual, and eidetic activations (Varela et al., 1991; Pöppel and Schill, 1995; Varela and Depraz, 2003):

[…] Imagination must necessarily correspond […] to a dynamic, emerging global pattern that is able both to integrate the body/brain activity at a large scale and subside rapidly, for the benefit of the next moment of mental life (Varela and Depraz, 2003, p. 201–202).

In the breadth of its dynamic aspects, Goethe's text preludes to the vision of imagination as a large-scale integration of multiple concurrent processes, as Varela and Depraz put out:

It is fair to say that imagination is emblematic, in fact, of a cluster of human abilities: imagining proper, or mental imagery, remembrance, fantasy, and dreaming. Imagination is an inexhaustible source in all these dimensions, explored and praised by human cultures throughout the world, a witness to its centrality (Varela and Depraz, 2003, p. 195).

In the pre-noetic dynamic and self-transforming shapelessness surrounding the Mothers, Goethe gives figuratively form to the pure potentiality of creative means (concepts, ideals, words, images), demonstrating their origin at the crossway of the faculty of intuition and symbolization. The whole process of imagination bases in fact on a continuous shift between the embodied and emotional experience in real life and its reuse and transformation in mental images:

[…]

All the Forms of Life

Float round your heads, active, not alive.

Whatever was, in all its glow and gleam,

Moves there still, since it must always be (6429–6432).

Mental imagery was thought to be symbolic and propositional in the past, while now, it is mostly considered as relying on the activation of neural sensorimotor areas, since it depends on the simulation of real visual and motor experiences. As Gallese claimed:

When indulging in visual or mental motor imagery, we reuse our visual or motor neural apparatus to imagine things and situations we are not actually perceiving or doing. Indeed when viewed from a neuroscientific perspective, the border separating real and imaginary worlds appears much less sharp and clear than what humans thought for centuries (Gallese, 2019, p. 117).

On the basis of the embodied simulation theory, language is able to create images out of physical experiences in the real world, compressing them at human scale in language. In Goethe's view, language morphology reflects the organic laws of nature, since both nature and language are ruled by the same principles: “Morphology is the doctrine of the shape, formation and transformation of organic living forms” (Goethe, 1985, 1.24, 365). Therefore, form, formation, and transformation characterize also language, which may reveal organic features. Furthermore, the principle of similarity applies to the three essential areas of human existence: the laws of nature, those of language, and of thought.

According to Louwerse (2008, 2011) language is both embodied and symbolic, as attested by the *Symbol Interdipendency Hypothesis*, and symbols are the basic tools of our imaginary world, being themselves embedded in human bodily experience. Words are able to “adhere”—as Goethe theorized”—to the objects of the real world. This does not mean that words are only referentially significant, but that thanks to their being imaginatively and rhetorically shaped and transformed, they are able to engage the invisible, immaterial essence of reality, whereby the meaning construction is powerfully guided by the permanent shift between speech imagery and embodied agency.

Two scenes after *Gloomy Gallery*, in *The Hall of the Knights, Dimly Lit*, Faust brings with him and shows on stage in front of the audience Helen and Paris, the prototypes of human beauty. Paris first and then Helen emerge out of a formless smoke bewildering the audience. At this point, Goethe creates a very peculiar textual strategy in order to represent the power of imagination active in the audience. To the reader's astonishment, Goethe does not describe the two characters. Instead, and more interestingly, he just reports the different comments and exclamations given by the audience in front of the unusual scene. This is the way in which Goethe makes it possible to let the audience see “what each desires, the marvelous” (6238). In this way, the author “performs” the imaginative act of reception: each person looking at the scene recognizes not a common principle of ideal beauty but the elements of their *own* concept of beauty. The inputs (the *eidola* of Helen and Paris) are recomposed and reconfigured at an imaginative level by the audience (metaphorically the reader), who is aware of the fictional nature of the representation. This process happens thanks to a phenomenon of gating, i.e., the selection of data derived from witnessing, together with a process of “naturalization” (Fludernik, 1996, 2009), i.e., the embodiment of prototypical structures and sequences, allowing the audience/reader to adapt and elaborate the performance in the text, experiencing in first person the fictional world, integrating the known subjective background with the new one provided by the theatrical scene. The act of reception is reported by Goethe giving voice to different characters of the audience (the old lady, the chamberlain, the learned man, etc.). Each of them comments on the physical appearance of the two mythological characters, but what they see is not univocal, and their comments refer to subjectively perceived different features and represent their contrasting meanings, like in the following lines:

[about Paris]

The Lady

Now sleep has overcome the charming boy.

The Chamberlain

And now he'll snore: that's natural, what joy! (6471–6472)

[about Helen]

An Older Lady

Tall, well formed, only the head is small.

A Younger Lady

Just look! Could clumsier feet exist at all? (6502–6503)

[…]

A Lady

How ugly, near that form so young and pure (6507).

Reporting the audience's reactions, Goethe meta-represents the process of aesthetic reception, taking place also during the reading act. In this way, Goethe completes the circular phenomenon of the imaginative process: the subject's imagination is triggered by the artist to fill in the ideal schema proposed by the text with the contents of his/her own experience, memories, engrams, and mnestic traces. In cognitive terms, the reader interacts with the fictional world through the creation of mental imagery on the basis of simulative processes, perceiving similarities and differences between the own experiences and emotions and those represented in the text. Therefore, in the literary, experience arises a sort of perceptual and emotional resonance with respect to the sensations, emotions, and actions narrated, due to the reader's activation of neural areas in charge of processing sensations, actions, and emotions in real life. It is not a case that the above-mentioned comments by the audience report sensual perceptions triggered by the appearance of Paris and Helen, like in the following comments about the ambrosia fragrance of youth:

A Young Lady

What refreshes my heart so deeply, that fragrance

Mixed with fumes from the burning incense?

An Older Lady

Truly! It's breath penetrates one's nature,

It comes from him! (6473–6476)

Therefore, the reader's mental imagery results from reactions to the suggestions evoked by the text put in conjunction with the personal experience: “Our relationship with fictional worlds is double-edged: on the one hand, we pretend them to be true, while, on the other, we are fully aware they are not.” (Gallese, 2011, p. 199).

The reproductive imagination of the reader is triggered by the dynamic nature of the images, on the basis of what we call Feeling of action (FoA), i.e., the bodily kinesthetic involvement of the reader, relying on the intensity of embodied simulation. We presume this to be stronger or weaker depending on the presence in the text of dynamic situations, actions, and motion, as well as dynamic images and rhetorical figures conveying movement and actions. This issue explains why many comments of the audience are referred to qualities of the body and to motion, like in the following example:

Another

He lifts his arm so lightly above his head (6465).

[…]

A Courtier

Soft and sly, she goes toward the sleeper (6506).

The sequence of comments by the audience is characterized in the whole by a climax of diverging impressions, which deny any real consistence of the figures as univocal *eidola*, scattered to the point of becoming disguised by the poet himself, letting the subjective imaginative elaboration of all inputs emerge.

## Conclusions

The aim of this study is to point out the dynamic emergent qualities of imagination as meta-represented in the literature. We highlighted how Goethe's evocation of the pre-noetic horizon of the Mothers' Kingdom and the evocation of Paris and Helen—this our thesis—are a poetical performative act that puts the audience in the play and the reader in the paradox condition of “assisting” to one of the most complex and still mysterious processes taking place in the human mind. The whole scene meta-represents the human activity presiding over mental imagery, considered not as abstract inner representations but as perceptual, sensuous, visual, auditory, and tactile. Goethe meta-represents the force of imagination in the dynamic figuration of the Mother's Kingdom as productive (creative) power, giving rise to what emerges out of the invisible and unstable. Moreover, he represents imagination as a circular process giving voice to the reproductive faculty raised in the receptive act. The presented scenes highlight the endogenous, dynamic, emergent qualities of imagination involving a cluster of cognitive faculties activated in order to construct meaning through the creation of aesthetic forms. In our opinion, these qualities are inherent and quintessential to the imagination as a cognitive and creative faculty. In this sense goes our claim to further studies in the field of a transdisciplinary venture inquiring the still uncharted aspects of the imagination as a circular phenomenon, which cannot be understood in abstract terms as a mind minor property but needs to be investigated as a reproductive and productive power with regard to both its creative and receptive aspects (RG).

## Data Availability Statement

The original contributions presented in the study are included in the article/supplementary material, further inquiries can be directed to the corresponding author/s.

## Author Contributions

This paper is the result of a common research of RG and GP. Both authors agree to be accountable for the content of the work.

## Conflict of Interest

The authors declare that the research was conducted in the absence of any commercial or financial relationships that could be construed as a potential conflict of interest.
